# Infected cyst in patients with autosomal dominant polycystic kidney disease: Analysis of computed tomographic and ultrasonographic imaging features

**DOI:** 10.1371/journal.pone.0207880

**Published:** 2018-12-05

**Authors:** Jiseon Oh, Cheong-Il Shin, Sang Youn Kim

**Affiliations:** 1 Department of Radiology, Seoul National University Hospital, Seoul, Korea; 2 Department of Radiology, Seoul National University College of Medicine, Seoul, Korea; Universita degli Studi di Bari Aldo Moro, ITALY

## Abstract

**Purpose:**

To investigate the imaging features of cyst infection in autosomal dominant polycystic kidney disease (ADPKD) patients using computed tomography (CT) and ultrasonography (US).

**Materials & methods:**

The institutional review board approved this retrospective study. Fifty-one episodes with proven cyst infection in forty-three ADPKD patients were included. Two experienced abdominal radiologists reviewed CT and US images and evaluated the following imaging features in consensus: cyst size, location, cyst shape, intracystic attenuation, intracystic echogenicity, intracystic heterogeneity, wall thickness, the presence of fluid-fluid level, septation, intracystic gas, pericystic fat infiltration, and pericystic hyperemia. Intracystic attenuation was measured for all infected cysts and two presumed normal cysts and compared using the Wilcoxon rank-sum test.

**Results:**

On CT scans, the median size of infected cysts was 5.5 cm (range: 2.3–18.8 cm) and 46 of 51 (90.2%) infected cysts were located in the subcapsular region. Most (48 of 51, 94.1%) infected cysts showed lobulated, focal bulging or irregular shape. Discernible wall thickening (84.1%) was the most frequently found imaging feature of infected cysts followed by relatively higher intracystic attenuation compared to normal cysts (79.1%) and pericystic fat infiltration (52.9%). Fluid/fluid level was found in 3 of 51 (5.9%) infected cysts and intracystic gas was found in 3 of 51 (5.9%) infected cysts, respectively. For hepatic cysts, 11 of 14 (78.6%) infected cysts showed pericystic hyperemia. Intracystic attenuation was significantly higher in infected cysts (median; 19.0 HU) than in presumed normal cysts (median; 8.5 HU) (P<0.001), and exceeded 25 HU in 18 (35.3%) of 51 infected cysts. Among the 41 infected cysts for which US images were available, 35 (85.1%) showed heterogeneous echogenicity.

**Conclusion:**

Minute imaging features such as minimal wall thickening or relatively high attenuation compared to normal cysts would be helpful to detect infected cysts in ADPKD patients.

## Introduction

Autosomal dominant polycystic kidney disease (ADPKD) is the most common hereditary renal disease, affecting 1 in 500 to 1 in 1,000 live births and 8–10% of patients with end-stage renal disease [1]. It is characterized by the development of numerous cysts in the kidney and liver parenchyma, which arise from various renal tubular segments and biliary ducts, which lead to increased kidney and liver size, respectively. Cysts are also associated with some of the most common complications of ADPKD such as intracystic hemorrhage, gross hematuria, obstruction mainly caused by liver cysts, and infections [2, 3]. Urinary tract infection, including cystic infection, is one of the major complications of polycystic kidney disease and occurs with an annual frequency of 0.01 episodes per patient [4]. Early diagnosis of cystic infection is clinically important, because cystic infection can be treated with antibiotics only if it is diagnosed early; in addition, the patient may die from sepsis if proper drainage is not performed in severe cases [5, 6].

The diagnosis of cyst infection is confirmed by extracting cyst fluid from the suspected cyst to help identify the microorganism or inflammatory findings such as neutrophils debris [4]. However, it is challenging to detect infected cysts in ADPKD patients with numerous cysts in the kidney or liver. Computed tomography (CT), which is a readily available and widely accessible cross-sectional imaging modality with a high spatial resolution, can be used. However, CT is believed to have a limited value in diagnosing infected cysts in patients with ADPKD because of a low sensitivity of less than 25% [7–9]. Positron emission tomography with fluorodeoxyglucose (FDG-PET) has recently been investigated as a promising diagnostic tool [8, 10–12] along with magnetic resonance imaging (MRI), especially diffusion-weighted imaging (DWI) as an alternative diagnostic modality for infected cysts [13, 14]. CT diagnostic criteria of enhanced wall thickening and/or perilesional inflammation that have been described in most previous studies are somewhat subjective and unclear [4, 7, 8, 10, 11]. In addition, imaging feature analysis of cyst infection in ADPKD patients using CT has rarely been performed. Therefore, we investigated the CT imaging features of cyst infection in ADPKD patients. The imaging features of ultrasonography (US) were also investigated in cases for which US data were available, given that US features of cyst infection have also not been reported in a case series or case-control studies.

## Materials and methods

### Patients

**Fig 1:**
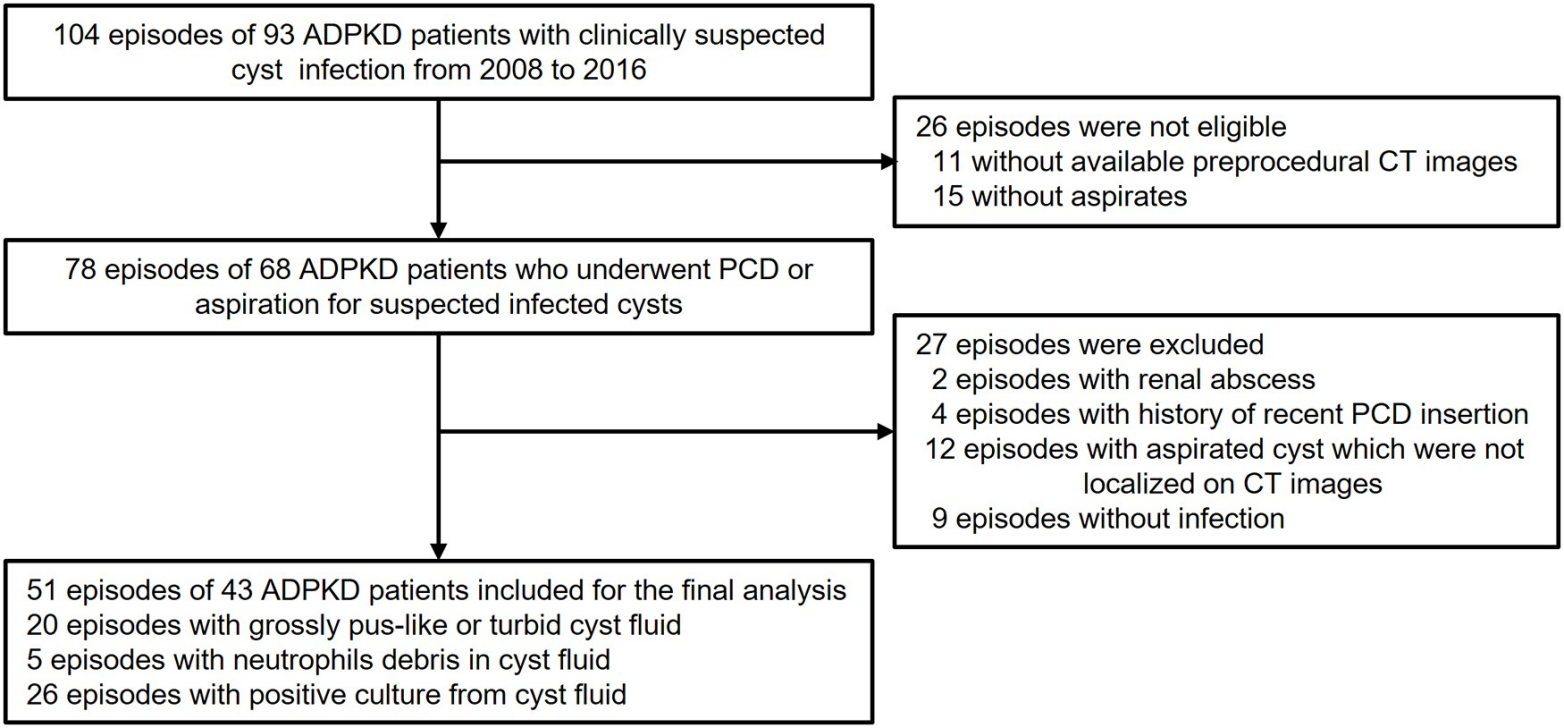
Flowchart for inclusion and exclusion of patients. Note: ADPKD = autosomal dominant polycystic kidney disease; PCD = Percutaneous catheter drainage.

The institutional review board approved this retrospective study (Seoul National University Hospital Institutional Review Board; H-1706-012-855), with a waiver of informed consent. From January 2008 to December 2016, 93 patients with ADPKD were admitted to the hospital for clinically suspected cyst infection (Fig 1). Among a total of 104 episodes, the episodes that met all three of the following criteria were included for CT features analysis in infected cysts: (1) the patient should have been undergone percutaneous catheter drainage (PCD) or aspiration for suspected infected cysts, (2) pre-procedural abdominal CT images should have been obtained less than 1 week before PCD or aspiration, and (3) histological reports of cyst fluid are available. Exclusion criteria as follows: (1) episodes with a history of recent PCD insertion within 2 weeks previously (n = 4), (2) episodes in which aspirated cysts were not localized on CT images (n = 12), (3) episodes of renal abscess (n = 2), and (4) episodes without fulfilment of the diagnostic criteria for infected cyst (n = 9).

Finally, 51 episodes of 43 ADPKD patients with proven cyst infection formed the study group for the CT feature analysis in infected cysts (12 men and 39 women; age 54.7 ± 10.4 years, age range 28–74 years). US images used as procedure guidance were available for 41 episodes in 37 patients. In addition, CT imaging features were assessed for the nine episodes excluded because aspirates showed no evidence of cyst infection; however, statistical analysis was not performed because of the small number of cases.

### Diagnosis of cyst infection

The diagnosis of cyst infection was established with the aspiration of cyst fluid and made in 1 of 3 ways: (1) cyst fluid showing complete pus-like or turbid features and/or (2) cyst fluid showing neutrophils debris and/or (3) cyst fluid showing microorganism [6]. Among 51 episodes with infected cysts, renal cysts and hepatic cysts comprised 31 and 20 episodes, respectively.

### Clinical variables

Patient medical records were reviewed; in addition, demographic and clinical data including age, sex, dialysis status, white blood cell (WBC) count, serum level of creatinine and C-reactive protein (CRP), glomerular filtration rate (GFR), and results from the cyst fluid/blood/urine culture study were collected.

The highest WBC count and serum CRP level within 1 week after the symptom onset were recorded. Serum creatinine level within 2 weeks before admission was recorded and the GFR was estimated by using the modified isotope dilution mass spectrometry (IDMS)-traceable 4-variable Modification of Diet in Renal Disease (MDRD) study equation. Blood and urine culture samples were cultured from all patients at the time of admission; however, some patients had already received antibiotics.

### Imaging protocol

All CT examinations were performed using multi-detector computed tomography (MDCT) scanners (Brilliance 64, Philips Medical Systems, Cleveland, Ohio; Aquilion ONE, Toshiba Medical Systems, Otawara, Japan; Sensation 16 Speed 4D, Siemens Medical Solutions, Forchheim, Germany). Scanning parameters for MDCT scanners included a gantry rotation time of 0.5 seconds, a pitch of 1.0 to 1.5, 150 to 230 mAs, 100 to 120 kVp, and a 512 x 512 matrix. The reconstruction parameters were a 3 mm slice thickness and a 2 mm or 3 mm reconstruction interval, both of which are standard for axial mages. Of the 51 episodes, 7 episodes have only pre-contrast images, 8 episodes have only portal venous phase images, and the remaining 36 cases have pre-contrast, arterial and portal venous/delayed images. Post-contrast CT scans were obtained after the administration of 1.5 mL/kg of nonionic contrast material with 350 mgI/mL for 30 seconds at a rate of 2.5 to 4.0 mL/s using a power injector. This injection was followed by a saline flush of 40 mL with the same injection rate as that of the contrast material. For arterial phase scanning, a 15-second delay was used after the attenuation of the descending aorta reached 100 Hounsfield Unit (HU) using bolus tracking. The portal venous phase was attained 35 seconds after the acquisition of the arterial phase or 70 seconds after the beginning of the contrast injection. Delayed phase (equilibrium phase) images were obtained 180 seconds after the start of contrast administration.

### Image analysis

CT and US images were assessed by a consensus reading of two radiologists with 11 years (C.I.S) and 12 years (S.Y.K) of experience in abdominal imaging.

On CT images, we evaluated the following imaging features: cyst size measured using maximum axial diameter; cyst shape classified into a round, lobulated, and focal bulging/irregular shape; and cyst location classified into subcapsular, parenchymal, and hilar. Intracystic attenuation (HU) of the infected cyst was also measured on pre-contrast or post-contrast images. To compare attenuation difference between an infected and normal cyst, attenuation was measured in two presumed normal cysts that showed no discernible wall, water attenuation, and no evidence of perilesional inflammation. In addition, we assessed whether each cyst appeared relatively hyperdense compared to surrounding normal cysts in the abdominal soft tissue window setting in a subjective manner but in consensus, and whether it showed heterogeneous attenuation. Pericystic hyperemia was evaluated only for hepatic cysts and defined as gross hyperenhancement outside of the cyst regardless of shape (wedge shape or circumferential) on arterial phase images. When a cyst wall was not imperceptible, it was considered to have discernible wall thickening, and in the 4× magnified image, wall thickness was measured three times and the largest measured value was recorded. We also evaluated whether each cyst showed fluid-fluid level, intracystic gas, and pericystic fat infiltration.

On US images, intracystic echogenicity was classified into anechoic versus echoic type. In terms of homogeneity, homogeneous echogenicity was defined as uniform intracystic echogenicity, and all other cases were considered heterogeneous echogenicity. We also evaluated whether each cyst showed fluid-fluid level and septation.

### Statistical analysis

Continuous variables are expressed as the mean ± standard deviation (SD) or median with range, according to the distribution of the data which was tested using the Shapiro–Wilk normality test. Categorical data are expressed as numbers (percentages). The attenuation of infected and normal cysts was compared using the Wilcoxon rank-sum test. Probability (P) value less than 0.05 was considered to indicate statistical significance. All statistical analyses were performed using software (MedCalc for Windows, version 17.6; MedCalc, Mariakerke, Belgium).

## Results

### Clinical characteristics

Among 51 episodes, 25 patients had 31 episodes of renal cyst infection, and 18 patients had 20 episodes of hepatic cyst infection. Median WBC count was 9,100/mm^3^ (range: 2,600–21,000/ mm^3^) and median serum level of CRP was 14.2 mg/dL (range: 2.6–35.0 mg/dL). Table 1 summarizes the demographic and clinical information.

**Table 1:** Clinical characteristics of 51 episodes of ADPKD patients with cyst infection.

	Episodes
Gender, n (%)	
Male	12 (23.5%)
Female	39 (76.5%)
Age (years)	
mean ± SD	54.7 ± 10.4
eGFR (ml/min per 1.73 m^2^)	
median (range)	20.7 (3.0–113.8)
Patients on Dialysis, n (%)	
Yes	32 (62.8%)
No	19 (37.2%)
WBC Count (x10^3^/mm^3^)	
median (range)	9.1 (2.6–21.7)
CRP (mg/dL)	
median (range)	14.2 (2.6–35.0)
Culture study of cystic fluid, n (%)	
Positive	24 (47.1%)
Negative	27 (52.9%)

Note: Continuous data expressed as the mean and SD (in case of the normal distribution) or the median and range (in case of violation of the normality assumption). Note: ADPKD = autosomal dominant polycystic kidney disease; SD = standard deviation; eGFR = estimated glomerular filtration rate; WBC = white blood cell; CRP = C-reactive protein

Microbiological documentation was available for 28 of 51 episodes (54.9%) (Table 2); positive cyst fluid culture was noted in 24 episodes, positive blood culture was noted in 13 episodes, and positive urine culture was noted in 17 episodes. *Escherichia coli* accounted for 18 (64.3%) of 28 retrieved bacterial strains. Cyst fluid culture was positive in all cases of 19 renal cyst infection except one. In fluid culture-negative case, blood and urine cultures were positive and the diagnosis of cyst infection was made because the aspirate was pus. There was no episode that was positive only in the urine culture. In addition, of the 9 episodes of hepatic cyst infection, 3 episodes were positive only in the blood culture (*Escherichia coli*, *Klebsiella pneumoniae*, *and Enterococcus faecium*), not in the cyst fluid. Seventeen percent of *Escherichia coli* (3 of 18) and twenty percent of *Klebsiella species* (1 of 5) were extended-spectrum beta-lactamase (ESBL)-producing strains. In addition, fifty percent of *Staphylococcus aureus* (one of two) and *Enterococcus species* (one of two) strains were resistant to methicillin and amoxicillin.

**Table 2:** Bacterial strains retrieved during 51 episodes of cyst infection in 43 patients with ADPKD.

Infection (episodes)	Positive culture		
	Cyst Fluid	Blood	Urine
Renal cyst infection (19 of 31 episodes, 61.3%)	Total (18 of 31 episodes, 58.1%)	Total (8 of 31 episodes, 25.8%)	Total (17 of 31 episodes, 54.8%)
	*Escherichia co*li (14) *Staphylococcus aureus* (1) *Enterococcus faecium* (1) *Klebsiella pneumoniae* (1) *Klebsiella oxytoca* (1)	*Escherichia coli* (6) * *Staphylococcus aureus* (1) *Enterococcus faecium* (1)	*Escherichia col*i (12) *Staphylococcus aureus* (1) * *Staphylococcus aureus* (1) *Enterococcus faecium* (1) *Klebsiella pneumoniae* (1) *Klebsiella oxytoca* (1)
Hepatic cyst infection (9 of 20 episodes, 45.0%)	Total (6 of 20 episodes, 30%)	Total (5 of 20 episodes, 25%)	
	*Escherichia coli* (3) *Klebsiella pneumoniae* (2) *Pseudomonas aeruginosa* (1)	*Escherichia coli* (1) *Klebsiella pneumoniae* (1) * *Escherichia coli* (1) * *Klebsiella pneumoniae* (1) * *Enterococcus faecium* (1)	

Note:

* The cyst fluid culture was negative in these episodes.

### CT imaging features

#### Morphologic evaluation

**Fig 2:**
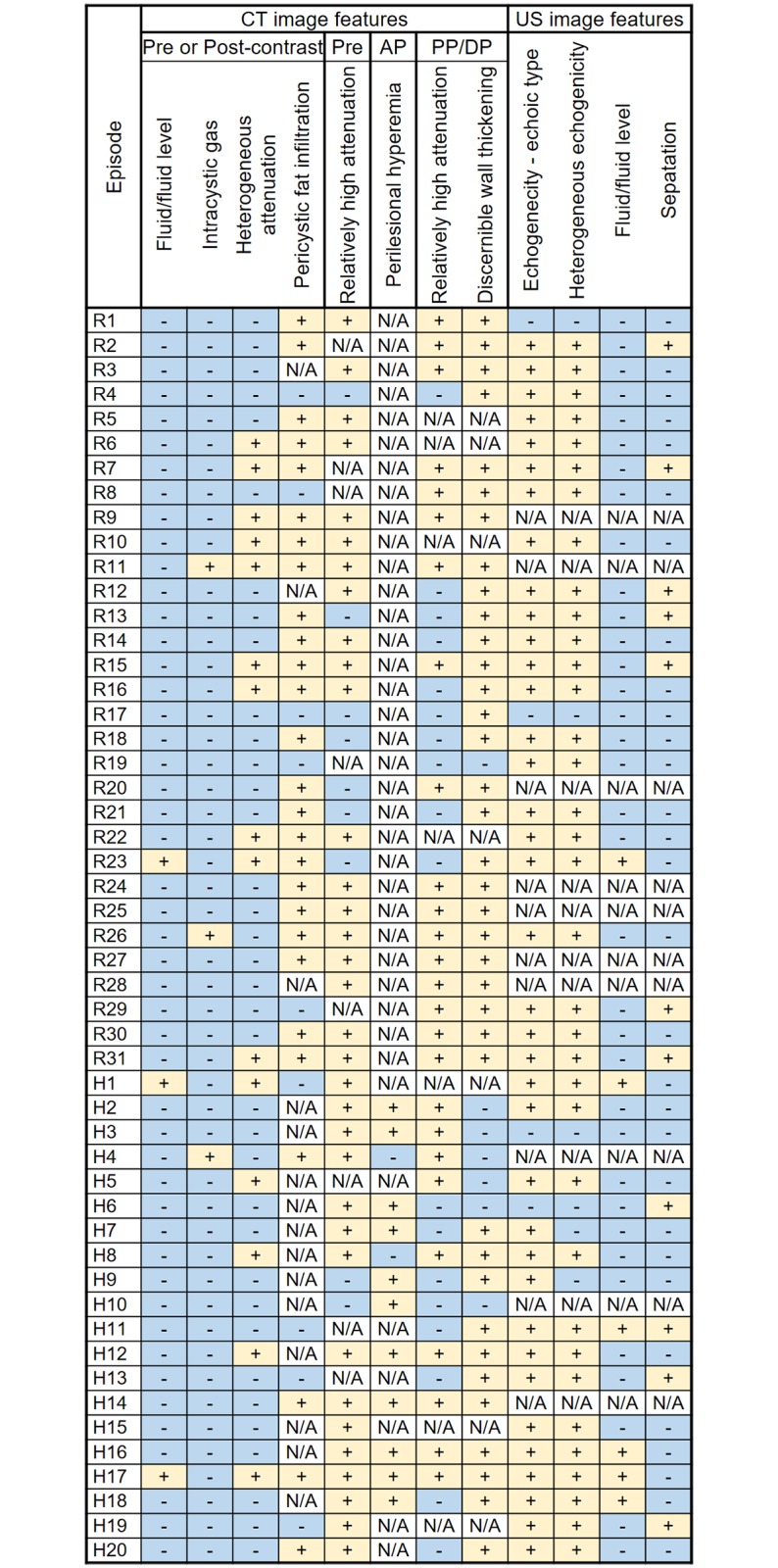
Mapping for CT imaging features in 51 episodes of cyst infection in 43 patients with ADPKD. Note: ADPKD = autosomal dominant polycystic kidney disease; Pre = pre-contrast images; AP = arterial phase images; PP = portal venous phase images; DP = delayed phase images; R, renal cyst; H, Hepatic cyst.

**Fig 3:**
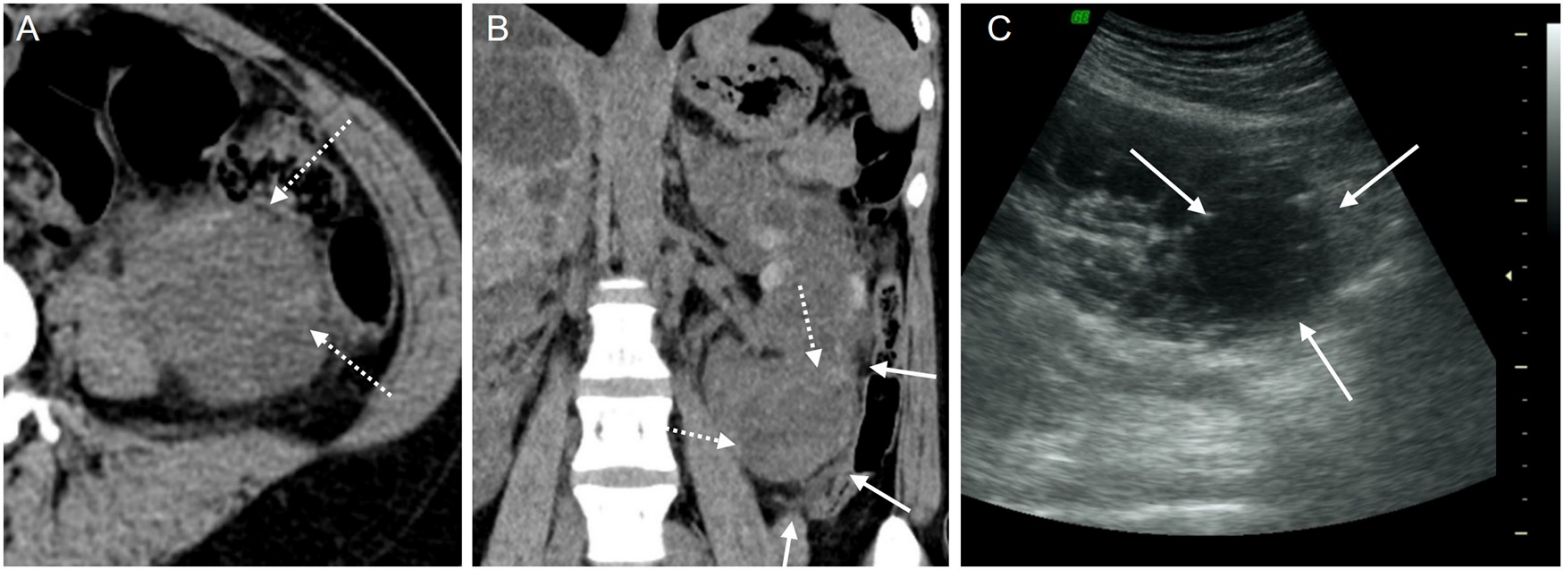
A 65-year-old female patient with an infected cyst (R10 in Fig 2). (A, B) Pre-contrast CT image shows a 4.5 cm sized cyst with a lobulated shape in left kidney lower pole (dotted arrows). The cyst has pericystic infiltration (arrows). (C) On US scan, the cyst (arrows) appears heterogeneous and echogenic.

Fig 2 and Table 3 summarize the CT findings. The median sizes of the infected renal and hepatic cysts were 5.5 cm (range: 2.9–11.6 cm) and 5.5 cm (range: 2.3–18.8 cm), respectively. Most infected cysts (30 of 31 renal cysts and 18 of 20 hepatic cysts) showed a lobulated, focal bulging or irregular shape. All 31 episodes of infected renal cysts and most of infected hepatic cysts (15 of 20 episodes, 75%) were located in the subcapsular region, and the remaining infected hepatic cysts (5 of 20 episodes, 25%) were located in the interstitial region. Fluid/fluid level was found in 3 of 51 (5.9%) infected cysts and intracystic gas was found in 3 of 51 (5.9%) infected cysts, respectively. In addition, 23 of 31 (74.2%) infected renal cysts and 4 of 20 (20%) infected hepatic cysts had pericystic fat infiltration (Fig 3).

**Table 3:** CT findings from 51 episodes of cyst infection in 43 patients with ADPKD.

Pre or Post-contrast	Available episodes, n	Renal cyst infection	Hepatic cyst infection	Total
		31	20	51
	Size (cm)			
	median (range)	5.5 (2.9–11.6)	5.5 (2.3–18.8)	5.5 (2.3–18.8)
	Shape, n (%)			
	Round	1 (3.2%)	2 (10%)	3 (5.9%)
	Lobulating	10 (32.3%)	11 (55%)	21 (41.2%)
	Focal bulging or irregular	20 (64.5%)	7 (35%)	27 (52.9%)
	Location, n (%)			
	Subcapsular	31 (100%)	15 (75%)	46 (90.2%)
	Interstitial	0 (0%)	5 (25%)	5 (9.8%)
	Fluid/fluid level, n (%)			
	Yes	1 (3.2%)	2 (10%)	3 (5.9%)
	No	30 (96.8%)	18 (90%)	48 (94.1%)
	Presence of intracystic gas, n (%)			
	Yes	2 (6.5%)	1 (5%)	3 (5.9%)
	No	29 (93.5%)	19 (95%)	48 (94.1%)
	Heterogeneous attenuation, n (%)			
	Yes	10 (32.3%)	5 (25%)	15 (29.4%)
	No	21 (67.7%)	15 (75%)	36 (70.6%)
	Presence of pericystic fat infiltration, n (%)			
	Yes	23 (74.2%)	4 (20%)	27 (52.9%)
	No	5 (16.1%)	4 (20%)	9 (17.6%)
	N/A	3 (9.7%)	12 (60%)	15 (29.4%)
Pre-contrast	Available episodes, n	Renal cyst infection	Hepatic cyst infection	Total
		26	17	43
	Intracystic attenuation (HU)			
	median (range)	19.0 (9–67)	19.0 (5–43)	19.0 (5–67)
	Relatively high attenuation, n (%)			
	Yes	19 (73.1%)	15 (88.2%)	34 (79.1%)
	No	7 (26.9%)	2 (11.8%)	9 (20.9%)
Arterial phase	Available episodes, n	Renal cyst infection	Hepatic cyst infection	Total
		22	14	36
	Perilesional hyperemia, n (%)			
	Yes	0 (0%)	11 (78.6%)	11 (30.6%)
	No	0 (0%)	2 (14.3%)	2 (5.5%)
	N/A	22 (100%)	1 (7.1%)	23 (63.9%)
Portal or delayed phase	Available episodes, n	Renal cyst infection	Hepatic cyst infection	Total
		27	17	44
	Intracystic attenuation (HU), n (%)			
	median (range)	22.0 (7–79)	20.0 (7–44)	21.0 (7–79)
	Relatively high attenuation, n (%)			
	Yes	17 (63.0%)	9 (52.9%)	26 (59.1%)
	No	10 (37.0%)	8 (47.1%)	18 (40.9%)
	Discernible wall thickening, n (%)			
	Yes	26 (96.3%)	11 (64.7%)	37 (84.1%)
	No	1 (3.7%)	6 (35.3%)	7 (15.9%)
	Wall thickness (mm)			
	median (range)	2.3 (1.5–3.9)	1.9 (1.5–3.3)	2.1 (1.5–3.9)

Note: Continuous data expressed as the mean and SD (in case of the normal distribution) or median and range (in case of violation of the normality assumption); HU = Hounsfield Unit. Note: Perilesional hyperemia is evaluated only in hepatic cyst. Note: Wall thickness is measured only when there is discernible wall thickening

#### Attenuation evaluation

**Fig 4:**
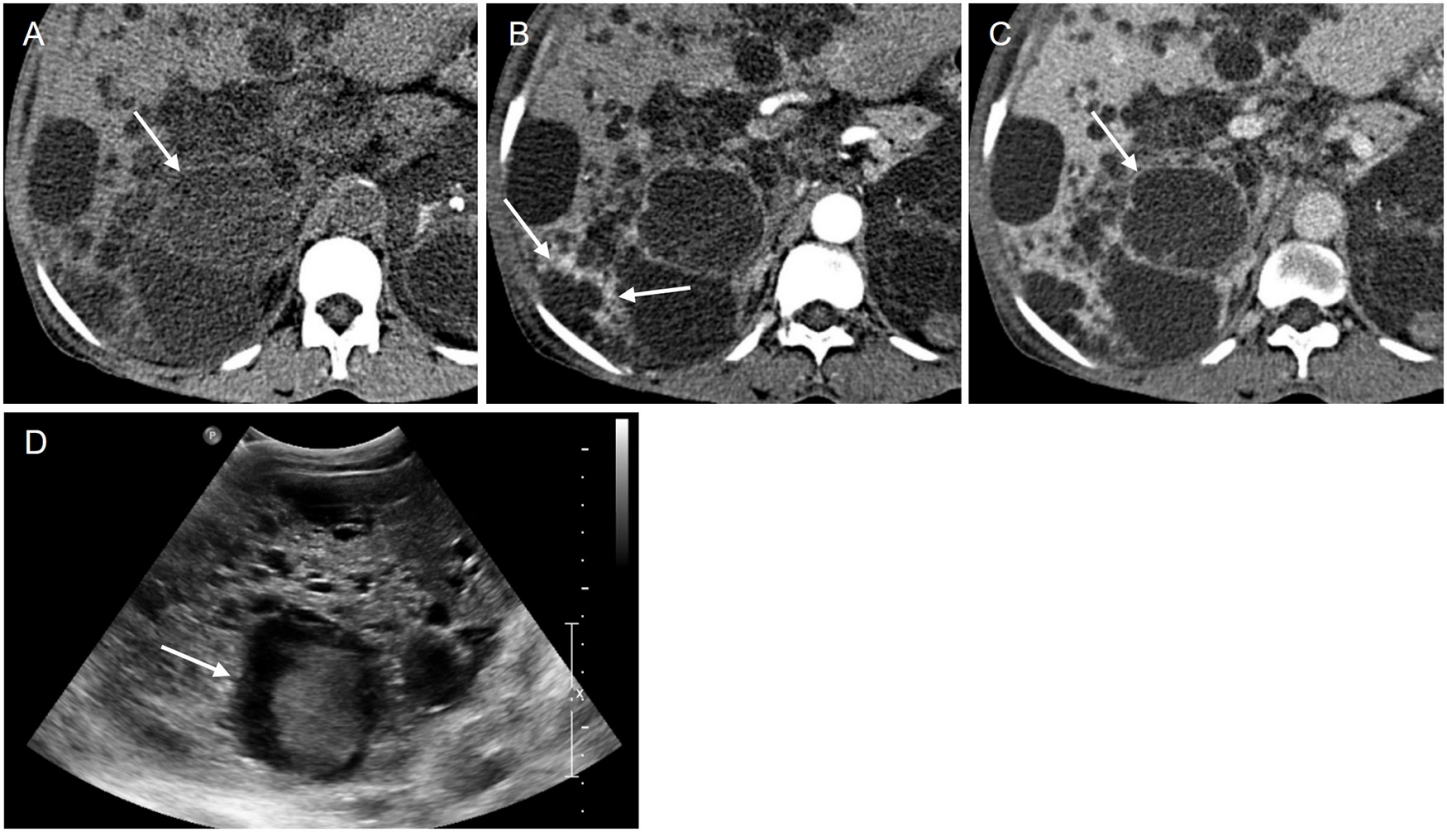
A 59-year-old female patient with an infected cyst (H12 in Fig 2). (A) The pre-contrast CT image shows a 5.5 cm sized cyst with a lobulated contour in segment 6 of the liver (arrow). The cyst appears to have a relatively high attenuation compared to the surrounding cysts. (B) The arterial phase image shows arterial hyperenhancement (arrows) in segment 6 of the liver; however, perilesional hyperemia could not be definitely diagnosed due to the intervening cysts between the lesion and hyperemia site. (C) The cyst shows discernible wall thickening in the portal venous phase. Of note, intracystic attenuation seems equal in contrast to the pre-contrast scan. (D) Ultrasonography shows a heterogeneous hyperechoic cyst with internal septation (arrow).

**Fig 5:**
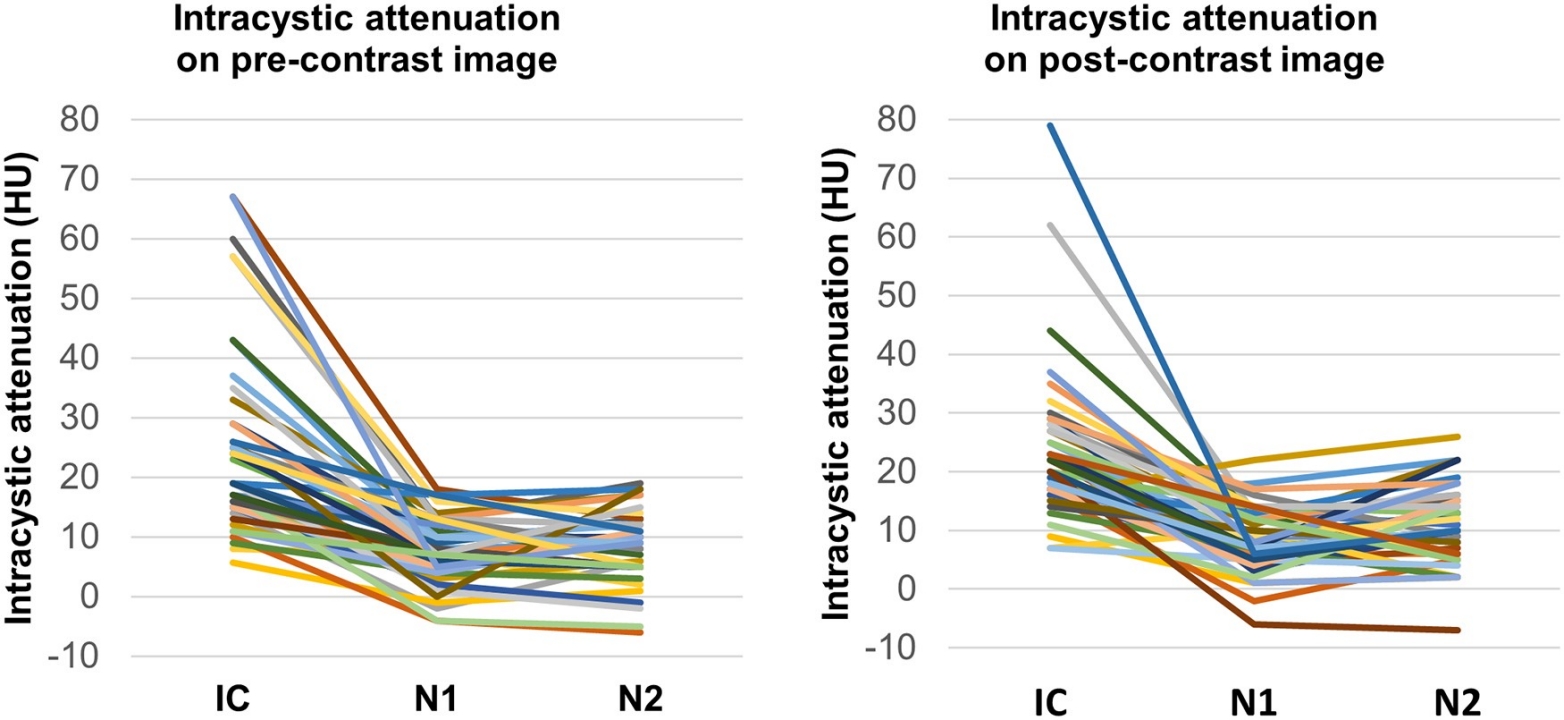
Intracystic attenuation (HU) of infected cysts and presumed normal cysts. (A) Intracystic attenuation was measured on pre-contrast images in 43 episodes. (B) Intracystic attenuation was measured on post-contrast images in 44 episodes. Note: HU = Hounsfield units; IC = infected cyst; N = presumed normal cyst.

The median attenuation of infected cysts was 19 HU (range: 5–67 HU) on pre-contrast images and 21 HU (range: 7–79 HU) on post-contrast images. Among the 51 episodes, intracystic attenuation exceeded 25 HU in 18 episodes (35.3%) and 50 HU in 4 episodes (7.8%). Infected cysts also appeared to have relatively high attenuation compared to the surrounding normal cysts in 34 of 43 episodes (79.1%) on pre-contrast images and 26 of 44 episodes (59.1%) on post-contrast images. Of the 36 infected cysts for which both pre-contrast and post-contrast images were available, 28 episodes matched each other and 7 and 1 episodes showed subjective higher attenuation than the surrounding normal cysts on only pre-contrast and post-contrast images, respectively (Fig 4). The median attenuation value of the 36 infected cysts was 18.0 HU and 19.5 HU in pre-contrast and post-contrast images, respectively. Measuring the attenuation difference between the infected cyst and presumed normal cyst revealed that intracystic attenuation was significantly higher in the infected cysts than in the presumed normal cysts for both pre-contrast and post-contrast images (Fig 5 and Table 4).

**Table 4:** Attenuation of infected cysts and presumed normal cysts.

Pre-contrast	Available episodes	Renal cyst (n = 26)	Hepatic cyst (n = 17)	Total (n = 43)
	Infected cysts (HU)			
	median (range)	19.0 (9–67)	19.0 (5–43)	19.0 (5–67)
	Presumed normal cysts (HU)			
	median (range)	8.8 (-5–17.5)	7.5 (-4.5–15.5)	8.5 (-5–17.5)
	Median difference			
	([95% CI], P-value)	15.0 ([10.5–25.8], P<0.001)	13.8 ([9.6–19.5], P<0.001)	14.5 ([11.3–19.5], P<0.001)
Post-contrast	Available episodes	Renal cyst (n = 27)	Hepatic cyst (n = 17)	Total (n = 44)
	Infected cysts (HU)			
	median (range)	22.0 (7–79)	20.0 (7–44)	21.0 (7–79)
	Presumed normal cysts (HU)			
	median (range)	9.5 (1.5–20)	9.5 (-6.5–24)	9.5 (-6.5–24)
	Median difference			
	([95% CI], P-value)	11.8 ([8.3–15.5], P<0.001)	12.0 ([6.2–17.0], P = 0.002)	12.0 ([9.3–14.8], P<0.001)

Note: CI = confidence interval

#### Enhancement-related evaluation

**Fig 6:**
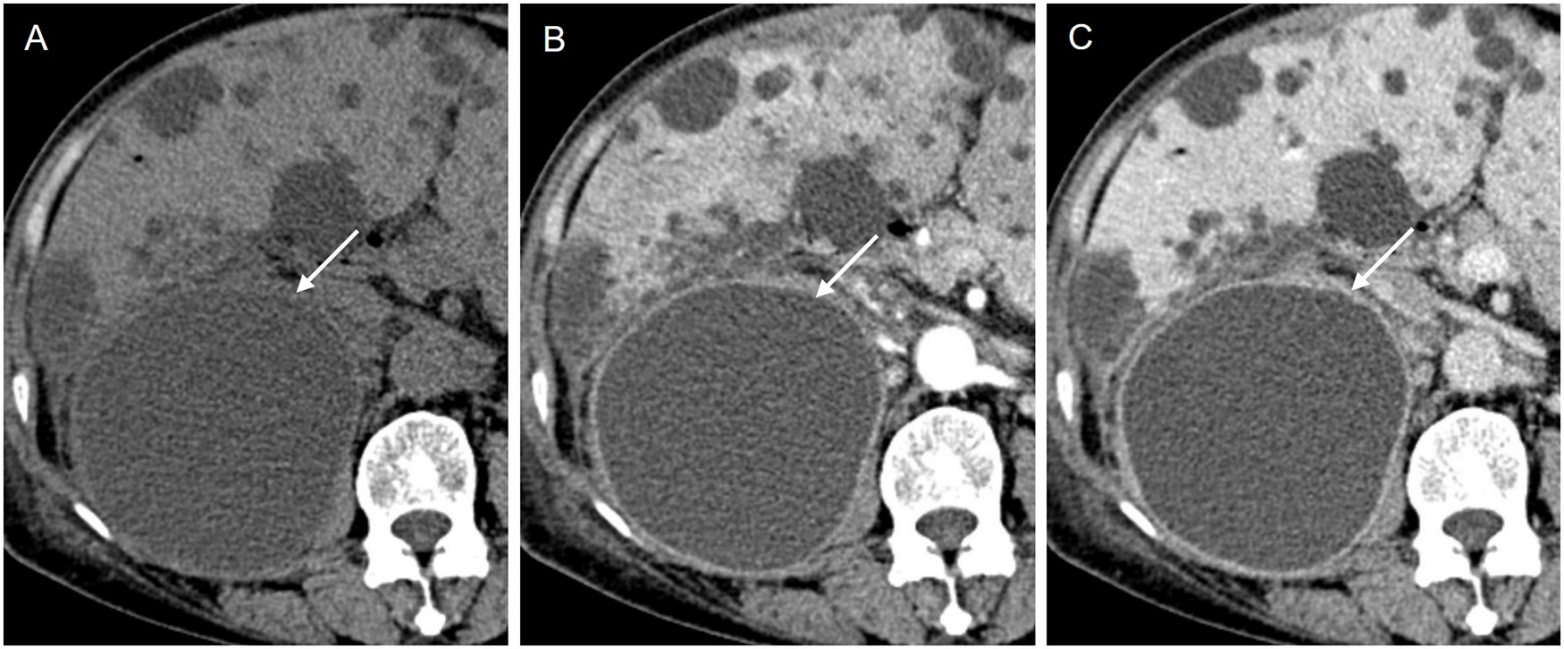
A 65-year-old female patient with an infected cyst (R24 in Fig 2). (A) Pre-contrast CT image shows a 9.7 cm sized round cyst that has higher attenuation than the surrounding cysts in right kidney upper pole (arrow). (B, C) On the arterial and portal venous phase images, the cyst shows discernible wall thickening with prominent wall enhancement and pericystic fat infiltration (arrows).

**Fig 7:**
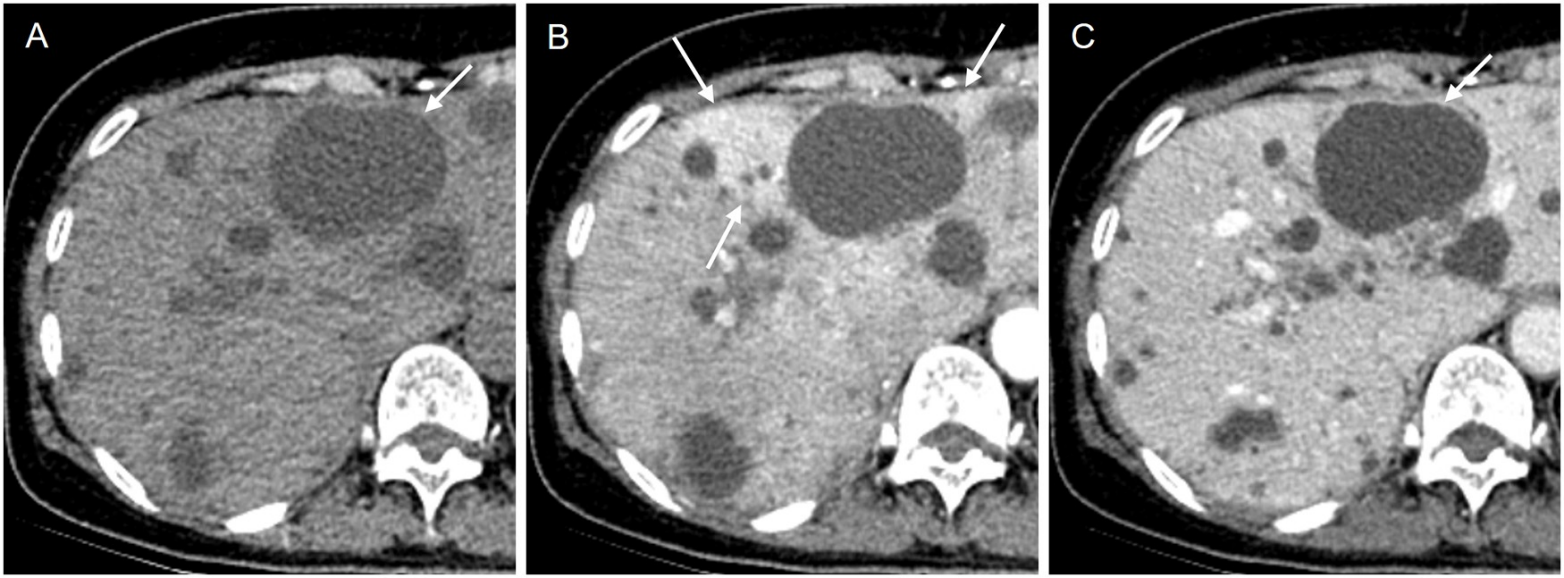
A 65-year-old female patient with an infected cyst (H10 in Fig 2). (A) On the pre-contrast CT image, a proven 6 cm infected cyst (arrow) shows no remarkable finding. (B) On the arterial phase image, there is perilesional hyperemia around the cyst (arrows). (B, C) On the arterial or delayed phase image, no discernible wall thickening is noted. In this case, arterial hyperemia is the only clue for cyst infection.

On post-contrast CT images, 37 of 51 (84.1%) infected cysts had discernible wall thickening with a median wall thickness of 2.1 mm (range: 1.5–3.9 mm) (Fig 6). All infected renal cysts except one showed discernible wall thickening, while 11 of 17 infected hepatic cysts delineated wall thickening. On arterial phase CT images, 11 of 14 infected hepatic cysts (78.6%) showed pericystic hyperemia (Fig 7).

#### Combined imaging feature

Forty-four of 51 (86.3%) episodes of infected cysts showed relatively higher attenuation on pre-contrast or post-contrast CT images. Among 14 infected hepatic cysts for which arterial phase images were available, 13 (92.9%) had perilesional hyperemia or pericystic fat infiltration. On post-contrast CT images, 37 of 44 (84.1%) infected cysts had discernible wall thickening, and 5 of the remaining 7 cases showed perilesional hyperemia or pericystic fat infiltration, and 6 of the remaining 7 cases showed relatively high attenuation than the surrounding normal cysts on pre-contrast and post-contrast images. In addition, 27 of 44 (61.4%) infected cysts had both discernible wall thickening and evidence of perilesional inflammation (i.e. perilesional hyperemia or pericystic fat infiltration) on post-contrast CT images, and 22 of 44 (50%) infected cysts had both discernible wall thickening and relatively higher than the surrounding normal cysts. Consequently, one case out of 51 episodes showed no evidence of cyst infection on CT, and in this case, cyst aspiration was performed at the site where the patient complained of tenderness.

#### CT findings of cysts without infection

**Fig 8:**
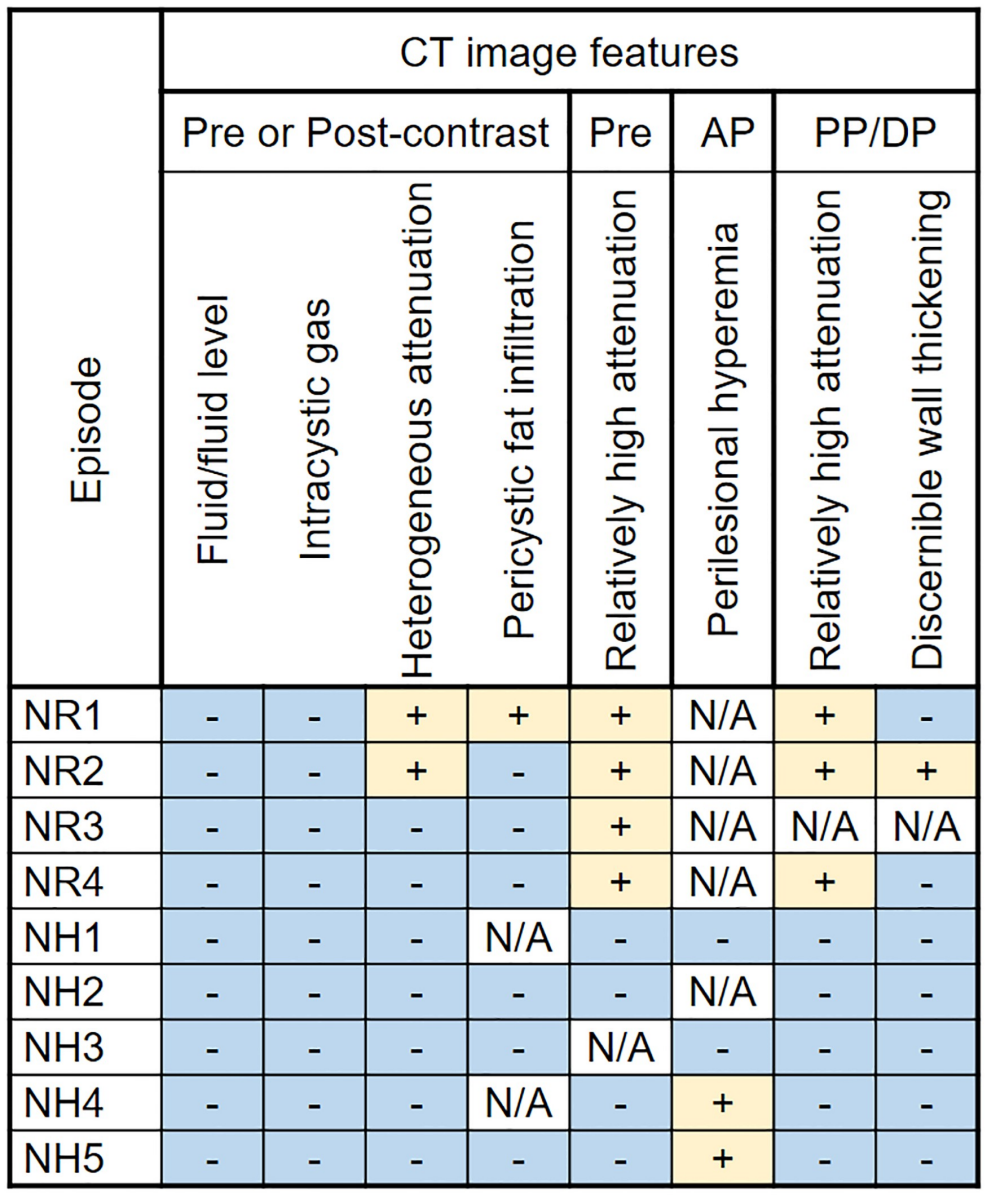
Mapping for CT imaging features in 9 episodes of noninfected cysts in 9 patients with ADPKD. Note: ADPKD = autosomal dominant polycystic kidney disease; Pre = pre-contrast images; AP = arterial phase images; PP = portal venous phase images; DP = delayed phase images; NR = noninfected renal cyst; NH = noninfected hepatic cyst.

There were 9 episodes from 7 patients that showed no evidence of cyst infection on aspirates. Among them, three episodes were acute hemorrhagic renal cysts with 69 HU, 61 HU, and 26 HU of intracystic attenuation, respectively, and one was a recent hemorrhagic renal cyst. Of the eight cysts for which we were able to assess wall thickness, only one (12.5%) with acute hematoma showed discernible wall thickening. CT findings were listed in Fig 8.

### US imaging features

Table 5 and Fig 2 summarize the US findings. Most infected cysts (22 of 24 renal cysts and 15 of 17 hepatic cysts) appeared hyperechoic and echogenic. When comparing intracystic heterogeneity on CT and US images, all 13 cysts showing heterogeneous attenuation on CT revealed heterogeneous echogenicity and 6 homogeneously attenuating cysts showed homogeneous echogenicity. Heterogeneous echogenicity was found in 22 homogeneously attenuating cysts; conversely, there was no homogeneously echoic cyst that showed heterogeneous attenuation. Fluid/fluid level and intracystic septation were found in 6 out of 41 (14.6%) and 11 out of 41 (26.8%) infected cysts, respectively.

**Table 5:** US findings from 41 episodes of cyst infection in 37 patients with ADPKD.

	Renal cyst infection (24 episodes)	Hepatic cyst infection (17 episodes)	Total (41 episodes)
Intracystic echogenicity, n (%)			
hypoechoic or hyperechoic	22 (91.7%)	15 (88.2%)	37 (90.2%)
anechoic	2 (8.3%)	2 (11.8%)	4 (9.8%)
Heterogeneous echogenicity, n (%)			
Yes	22 (91.7%)	13 (76.5%)	35 (85.4%)
No	2 (8.3%)	4 (23.5%)	6 (14.6%)
Fluid-fluid level, n (%)			
Yes	1 (4.2%)	5 (29.4%)	6 (14.6%)
No	23 (95.8%)	12 (70.6%)	35 (85.4%)
Septation, n (%)			
Yes	7 (29.2%)	4 (23.5%)	11 (26.8%)
No	17 (70.8%)	13 (76.5%)	30 (73.2%)

## Discussion

The most common CT feature for infected cysts in ADPKD in this study was discernible wall thickening, which was observed in 84.1% of cases; this has also been regarded as a suggestive finding for infected cysts in previously reported studies. The median wall thickness of infected cysts was 2.1 mm, ranging from 1.5 mm to 3.9 mm; therefore, a definition of wall thickening ≥3 mm used in a previously reported study [15], might be misleading considering our results. Discernible wall thickening was found to be more common in renal cyst infection (96.3%) than in hepatic cyst infection (64.7%). One possible explanation would be that the liver in patients with ADPKD often retains cyst-free parenchyma, whereas in most cases, the kidney is completely replaced by cysts, so the wall of hepatic cysts may be less perceptible because the tissue contrast between the cyst wall and the surrounding liver parenchyma would be lower than the tissue contrast between the renal cyst and adjacent cysts.

Sallée et al.[6] proposed that cyst infection is a probable diagnosis in the concurrent manifestation of four conditions: fever (temperature >38°C for >3 days), abdominal tenderness in the kidney or liver area, increased level of C-reactive protein (CRP) (>5 mg/dL) and the absence of CT augmentation for recent intracystic bleeding. A previous study, which compared clinical features of cyst infection and hemorrhage in AKPKD patients, defined the cutoff value for intracystic attenuation to distinguish cyst hemorrhage from infected cysts as 25 HU [15]. However, 25 HU cannot be the threshold for differentiating hemorrhagic cysts from infected cysts considering the fact that our results showed that the intracystic attenuation of the infected cysts had a wide range from 5 HU to 67 HU with a median value of 19.0 HU. Moreover, some infected cysts showed attenuation greater than 50 HU, strongly suggesting an acute hemorrhagic cyst, so even if the cyst appears as an acute hemorrhagic cyst, the possibility of combined infection cannot be ruled out.

With regard to intracystic attenuation, a relatively high attenuation based on the visual perception by the reviewer was a common feature in infected cysts. Interestingly, it was more frequently noted in the pre-contrast scan than in the post-contrast scan, although the mean attenuation of the cyst was slightly higher in the post-contrast scan. This phenomenon was interpreted to be because a subtle attenuation difference can be better recognized in pre-contrast images than in post-contrast images, which have a wider window width and higher window level than pre-contrast images due to enhanced high-attenuating parenchyma of the liver or kidneys.

Perilesional hyperemia is one of the signs of inflammation. Arterial phase scans sometimes provide diagnostic clues, because perilesional hyperemia around a certain cyst could be the only finding indicating infected hepatic cyst. In addition, the post-contrast CT scan would be recommended in the diagnosis of an infected cyst if a patient is not at risk for contrast-induced nephropathy, because discernible wall thickening is a common feature that can often be assessed on enhanced images. However, the pre-contrast scan also has value because several features, such as relatively high or heterogeneous attenuation and the presence of intracystic gas, can be evaluable on CT without enhancement.

In our study, 22 infected cysts with homogeneous attenuation on CT showed heterogeneous echogenicity on US, indicating that US can demonstrate intracystic complicated fluid content more sensitively. Although not investigated in this study, US also has advantages of no radiation hazard and a crude evaluation of vascularity without contrast enhancement. However, it would be difficult to detect a complicated cyst using US as a first imaging modality because kidney or liver affected by ADPKD is usually markedly enlarged, sometimes measuring more than 20 cm in size.

Most infected cysts were shown to be located in the subcapsular region. Rather than being an indication that infected cysts occur more frequently in the subcapsular region, this observation reflects that cysts in the subcapsular location could be more easily aspirated than deeply seated cysts.

CT features of infected cysts investigated in this study are not specific and could be seen in hemorrhagic cysts or infrequently in cysts with sterile inflammation. Case-control study design would be better to assess the diagnostic performance of CT features; however, the number of non-infected cysts was thought to be too small for statistical analysis and this is a major limitation of our study. However, the problem of CT diagnosis for infected cysts has been low sensitivity rather than low specificity; therefore, we think that it is notable that all infected cysts, except one lesion, showed at least one positive finding in the CT features. Discernible wall thickening, the most frequent CT feature, was rarely noted in non-infected cysts; therefore, if there is a cyst showing discernible wall thickening among numerous cysts on CT scan, careful investigation would be helpful to detect an infected cyst and may reduce the need for high cost diagnostic modality. Nevertheless, the matter of specificity in imaging features drawn in this study still remains complicating confident clinical application, so further studies with a control group are warranted to prove or disprove the usefulness of these imaging features in the diagnosis of infected cysts in ADPKD patients.

There were several other limitations in this study. First, there was a substantial number of cases that were excluded, and this could result in a bias; approximately 15% (12/78) were excluded because aspirated cysts were not localized on CT due to lack of a guiding US image or no written record about the aspiration site. This problem was an inherent limitation in this retrospective study. Second, there was no comparison with the emerging promising diagnostic modalities of FDG-PET or MRI. Therefore, further studies with a larger number of cases including a control group are warranted to evaluate the diagnostic performance of CT, hopefully with a comparison to FDG-PET or MRI. Third, for patients with ADPKD, the use of contrast media can be a problem although much information was obtained from the post-contrast scan. In our study group, 32 of 51 episodes (62.8%) were cases of ADPKD patients on dialysis, which allowed us to perform contrast-enhanced CT. However, considering that a larger proportion of ADPKD cases progress to ESRD compared to those with other forms of chronic kidney disease [16, 17], it is clinically challenging to perform contrast-enhanced CT in all APDKD patients with suspected cyst infection.

In conclusion, minute findings such as minimal wall thickening, pericystic fat infiltration, or relatively higher attenuation compared to normal cysts could be suggestive of infected cyst in ADPKD patients. In hepatic cysts, perilesional hyperemia may be the only finding suggestive of cyst infection. Therefore, it will be helpful to evaluate CT or US imaging features more carefully and comprehensively if a patient with ADPKD has symptoms that raise a suspicion of cyst infection.

## Abbreviations

ADPKD: Autosomal dominant polycystic kidney disease
CRP: C-reactive protein
CT: computed tomography
GFR: glomerular filtration rate
HU: Hounsfield units
PCD: percutaneous catheter drainage
US: ultrasonography
WBC: white blood cell

## References

[1] Torres VE, Harris PC, Pirson Y. Autosomal dominant polycystic kidney disease. Lancet (London, England). 2007;369(9569):1287–301. Epub 2007/04/17. 10.1016/s0140-6736(07)60601-1 . 17434405 10.1016/S0140-6736(07)60601-1

[2] Grantham JJ. Clinical practice. Autosomal dominant polycystic kidney disease. The New England journal of medicine. 2008;359(14):1477–85. Epub 2008/10/04. 10.1056/NEJMcp0804458 . 18832246 10.1056/NEJMcp0804458

[3] Christophe JL, van Ypersele de Strihou C, Pirson Y. Complications of autosomal dominant polycystic kidney disease in 50 haemodialysed patients. A case-control study. The U.C.L. Collaborative Group. Nephrology, dialysis, transplantation: official publication of the European Dialysis and Transplant Association—European Renal Association. 1996;11(7):1271–6. Epub 1996/07/01. . 8672022

[4] Jouret F, Lhommel R, Devuyst O, Annet L, Pirson Y, Hassoun Z, et al Diagnosis of cyst infection in patients with autosomal dominant polycystic kidney disease: attributes and limitations of the current modalities. Nephrology, dialysis, transplantation: official publication of the European Dialysis and Transplant Association—European Renal Association. 2012;27(10):3746–51. Epub 2012/11/02. 10.1093/ndt/gfs352 . 23114901 10.1093/ndt/gfs352

[5] Schwab SJ, Bander SJ, Klahr S. Renal infection in autosomal dominant polycystic kidney disease. The American journal of medicine. 1987;82(4):714–8. Epub 1987/04/01. . 3565428 10.1016/0002-9343(87)90005-2

[6] Sallee M, Rafat C, Zahar JR, Paulmier B, Grunfeld JP, Knebelmann B, et al Cyst infections in patients with autosomal dominant polycystic kidney disease. Clinical journal of the American Society of Nephrology: CJASN. 2009;4(7):1183–9. Epub 2009/05/28. 10.2215/CJN.01870309 . 19470662 10.2215/CJN.01870309 PMC2709515

[7] Neuville M, Hustinx R, Jacques J, Krzesinski JM, Jouret F. Diagnostic Algorithm in the Management of Acute Febrile Abdomen in Patients with Autosomal Dominant Polycystic Kidney Disease. PloS one. 2016;11(8):e0161277 Epub 2016/08/17. 10.1371/journal.pone.0161277 . 27529555 10.1371/journal.pone.0161277 PMC4987061

[8] Bobot M, Ghez C, Gondouin B, Sallee M, Fournier PE, Burtey S, et al Diagnostic performance of [(18)F]fluorodeoxyglucose positron emission tomography-computed tomography in cyst infection in patients with autosomal dominant polycystic kidney disease. Clinical microbiology and infection: the official publication of the European Society of Clinical Microbiology and Infectious Diseases. 2016;22(1):71–7. Epub 2015/10/11. 10.1016/j.cmi.2015.09.024 . 26454062 10.1016/j.cmi.2015.09.024

[9] Balbo BE, Sapienza MT, Ono CR, Jayanthi SK, Dettoni JB, Castro I, et al Cyst infection in hospital-admitted autosomal dominant polycystic kidney disease patients is predominantly multifocal and associated with kidney and liver volume. Brazilian journal of medical and biological research = Revista brasileira de pesquisas medicas e biologicas. 2014;47(7):584–93. Epub 2014/06/12. 10.1590/1414-431X20143584 . 24919173 10.1590/1414-431X20143584 PMC4123838

[10] Kwon HW, Lee HY, Hwang YH, Park HC, Ahn C, Kang KW. Diagnostic performance of 18F-FDG-labeled white blood cell PET/CT for cyst infection in patients with autosomal dominant polycystic kidney disease: a prospective study. Nuclear medicine communications. 2016;37(5):493–8. Epub 2016/03/26. 10.1097/MNM.0000000000000466 . 27014954 10.1097/MNM.0000000000000466

[11] Piccoli GB, Arena V, Consiglio V, Deagostini MC, Pelosi E, Douroukas A, et al Positron emission tomography in the diagnostic pathway for intracystic infection in adpkd and "cystic" kidneys. a case series. BMC nephrology. 2011;12:48 Epub 2011/10/01. 10.1186/1471-2369-12-48 . 21957932 10.1186/1471-2369-12-48 PMC3197475

[12] Jouret F, Lhommel R, Beguin C, Devuyst O, Pirson Y, Hassoun Z, et al Positron-emission computed tomography in cyst infection diagnosis in patients with autosomal dominant polycystic kidney disease. Clinical journal of the American Society of Nephrology: CJASN. 2011;6(7):1644–50. Epub 2011/06/28. 10.2215/CJN.06900810 . 21700816 10.2215/CJN.06900810

[13] Ichioka K, Saito R, Matsui Y, Terai A. Diffusion-weighted magnetic resonance imaging of infected renal cysts in a patient with polycystic kidney disease. Urology. 2007;70(6):1219 Epub 2007/12/26. 10.1016/j.urology.2007.09.040 . 18158052 10.1016/j.urology.2007.09.040

[14] Katano K, Kakuchi Y, Nakashima A, Takahashi S, Kawano M. Efficacy of diffusion-weighted magnetic resonance imaging in detecting infected cysts in a case of polycystic kidney disease. Clinical nephrology. 2011;75 Suppl 1:24–6. Epub 2011/01/29. . 21269589 10.5414/cn106439

[15] Suwabe T, Ubara Y, Sumida K, Hayami N, Hiramatsu R, Yamanouchi M, et al Clinical features of cyst infection and hemorrhage in ADPKD: new diagnostic criteria. Clinical and experimental nephrology. 2012;16(6):892–902. Epub 2012/06/13. 10.1007/s10157-012-0650-2 . 22688273 10.1007/s10157-012-0650-2

[16] Zhang QL, Rothenbacher D. Prevalence of chronic kidney disease in population-based studies: systematic review. BMC public health. 2008;8:117 Epub 2008/04/15. 10.1186/1471-2458-8-117 . 18405348 10.1186/1471-2458-8-117 PMC2377260

[17] Gilbertson DT, Liu J, Xue JL, Louis TA, Solid CA, Ebben JP, et al Projecting the number of patients with end-stage renal disease in the United States to the year 2015. Journal of the American Society of Nephrology: JASN. 2005;16(12):3736–41. Epub 2005/11/04. 10.1681/ASN.2005010112 . 16267160 10.1681/ASN.2005010112

